# New fecal bacterial signature for colorectal cancer screening reduces the fecal immunochemical test false-positive rate in a screening population

**DOI:** 10.1371/journal.pone.0243158

**Published:** 2020-12-01

**Authors:** Marta Malagón, Sara Ramió-Pujol, Marta Serrano, Joan Amoedo, Lia Oliver, Anna Bahí, Josep Oriol Miquel-Cusachs, Manel Ramirez, Xavier Queralt-Moles, Pau Gilabert, Joan Saló, Jordi Guardiola, Virginia Piñol, Mariona Serra-Pagès, Antoni Castells, Xavier Aldeguer, L. Jesús Garcia-Gil

**Affiliations:** 1 GoodGut SL, Girona, Spain; 2 Institut d’Investigació Biomèdica de Girona (IDIBGI), Salt, Spain; 3 Universitat de Girona, Girona, Spain; 4 Consorci Hospitalari de Vic, Vic, Spain; 5 Laboratori Clínic Territorial de Girona (LCTG), Salt, Spain; 6 Hospital Universitari de Bellvitge (IDIBELL), l’Hospitalet de Llobregat, Spain; 7 Hospital Universitari de Girona Dr. Josep Trueta, Girona, Spain; 8 Gastroenterology Department, Hospital Clínic, Institut d’Investigacions Biomèdiques August Pi i Sunyer (IDIBAPS), University of Barcelona, CIBERehd, Barcelona, Spain; University of Minnesota Twin Cities, UNITED STATES

## Abstract

Guidelines recommend routine screening for colorectal cancer (CRC) in asymptomatic adults starting at age 50. The most extensively used noninvasive test for CRC screening is the fecal immunochemical test (FIT), which has an overall sensitivity for CRC of approximately 61.0%-91.0%, which drops to 27.0%-67.0% for advanced adenomas. These figures contain a high false-positive rate and a low positive predictive value. This work aimed to develop a new, noninvasive CRC screening tool based on fecal bacterial markers capable of decreasing FIT false-positive rates in a FIT-positive population. We defined a fecal bacterial signature (*RAID-CRC Screen*) in a proof-of-concept with 172 FIT-positive individuals and validated the obtained results on an external cohort of 327 FIT-positive subjects. All study participants had joined the national CRC screening program. In the clinical validation of *RAID-CRC Screen*, a sensitivity of 83.9% and a specificity of 16.3% were obtained for the detection of advanced neoplasm lesions (advanced adenomas and/or CRC). FIT 20 μg/g produced 184 false-positive results. Using *RAID-CRC Screen*, this value was reduced to 154, thus reducing the false-positive rate by 16.3%. The *RAID-CRC Screen* test could be implemented in CRC screening programs to allow a significant reduction in the number of colonoscopies performed unnecessarily for FIT-positive participants of CRC screening programs.

## Introduction

Colorectal cancer (CRC) is the second leading cause of cancer death worldwide, accounting for more than 880,000 deaths in 2018 [1]. The highest incidence rates are found in the most developed countries (i.e., Australia, New Zealand, and countries in Europe and North America), while the lowest rates are found in the poorest countries (i.e., those in Africa and Southcentral Asia). These geographic differences could be associated with Western diets and other environmental aspects. Low socioeconomic status is also related to a higher risk for developing CRC [2].

CRC mortality rates have declined progressively in many Western countries as governments have begun focusing their efforts at different prevention levels. Primary prevention aims to decrease the risk of developing colorectal neoplasms by following a healthy diet and lifestyle and chemoprevention [3]. CRC screening is a secondary prevention strategy for reducing incidence and mortality by detecting and removing precursor lesions such as colorectal adenomas [4]. A tertiary prevention strategy is CRC surveillance, which seeks to minimize the impact of previously established colorectal neoplasms on the prognosis of the patient [5].

CRC is a good candidate for disease screening, as it constitutes a significant health issue, its natural history has been widely studied, diagnostic methods for its early detection are available and its treatment is more effective when it is diagnosed early [6]. Moreover, screening programs for CRC have been demonstrated to be more cost-effective than other screening programs, such as those for breast and cervix cancers [7]. Improvement in screening quality and adherence, however, is pivotal for further reducing the prevalence of CRC-associated death. The most extensively used CRC screening strategies are based on endoscopic examinations (i.e., colonoscopy and flexible sigmoidoscopy) and fecal tests (i.e., the guaiac-based fecal occult blood test and fecal immunochemical test (FIT)). Colonoscopy is currently used as a CRC screening method in countries such as Germany and Poland. Although its sensitivity and specificity for advanced neoplasia (AN; i.e., advanced adenoma (AA) and/or CRC) approaches 100% in experienced hands [8], it also has some limitations: it requires exhaustive bowel cleansing and is time-consuming, painful, and expensive [9]. To overcome these limitations, there is an increasing interest in the use of a two-step screening strategy by first using noninvasive screening and then after a positive result, performing the colonoscopy. The most commonly used noninvasive method is FIT, which specifically detects human hemoglobin in feces. The quantitative nature of FIT allows the selection of an optimal cut-off concentration for the desired target population.

Different screening programs have been implemented in regions of Spain for those aged 50-69 years. Most of these programs first use FIT (cut-off at 20 μg of hemoglobin/g of feces), followed by a colonoscopy when a positive FIT result is obtained. In the Catalonia region, in 2013, 47.8% of the invited population agreed to participate in the CRC screening program, of which 5.4% tested positive [10].

Data from 19 studies in which FIT performance in CRC screening was assessed showed that the overall sensitivity and specificity for CRC were 79.0% and 94.0%, respectively [11]. Importantly, CRC screening strategies need to show high performance in detecting not only CRC but also precancerous lesions. In this setting, FIT sensitivity for AAs has been shown to be 28.0%, while its positive predictive value (PPV) has been shown to be 13.3%, which results in a high false-positive rate for AN [12]. False-positive results lead to patient concerns and unnecessary colonoscopies, with associated potential risks and costs. Therefore, in order to reduce false-positive results, it is necessary to develop new noninvasive tools for CRC screening with higher specificity.

In recent years, growing attention has been given to the role of microbiota in carcinogenesis. Microorganisms are thought to be involved in up to 20.0% of cancers [13], specifically colorectal cancer [14]. The intestinal mucosa is constantly exposed to the gut microbiota and its derived metabolites, which have been observed to stimulate the immune response with the potential to cause inflammation [15]. Several research groups have sequenced the 16S rRNA genes of bacteria from colonic mucosa or feces and reported that patients with CRC show colorectal dysbiosis [16–19]. Some of the identified species are suspected to be involved in colorectal tumorigenesis. These species include *Gemella morbillorum* (GMLL), *Peptostreptococcus stomatis* (PTST), and *Bacteroides fragilis* (BCTF). All these data suggest that the fecal gut microbiota is a potential alternative noninvasive tool for CRC screening. From these studies, we designed quantitative polymerase chain reaction (qPCR) systems that specifically target those bacterial markers. Moreover, we have also studied other bacterial indicators retrieved from other studies and previously developed by our group (i.e., B10, best BLAST match *Faecalibacterium prausnitzii* (FPRA); B46, best BLAST match *Subdoligranulum variabile*; and B48, best BLAST match *Ruminococcus* sp., *Roseburia* sp., and *Coprococcus* sp.) [20,21].

In this work, we developed a new, noninvasive tool for CRC screening (i.e., *RAID-CRC Screen*) based on a fecal bacterial signature that complements FIT and increases its specificity and PPV for AN detection among FIT-positive participants. Proof-of-concept and clinical validation of the designed tool in two independent cohorts of an organized, population-based CRC screening program are reported.

## Methods

### Study population

Participants of the Catalan CRC screening program were invited to participate in the study. The inclusion criteria were as follows: (1) asymptomatic subjects; (2) subjects aged 50–69 years (both included); and (3) subjects with a positive FIT result (cut-off at 20 μg of hemoglobin/g of feces). The exclusion criteria were as follows: (1) subjects who had received antibiotics the month prior to inclusion; (2) subjects who had received chemotherapy and/or radiotherapy the last 6 months prior to inclusion; (3) subjects with severe comorbidity that, in the opinion of the investigator, should preclude participation in the study; and (4) subjects who were pregnant at the time of inclusion.

In the proof-of-concept study, a cohort consisting of 189 consecutive FIT-positive participants in the Catalan CRC screening program was recruited (Table 1). The recruiting centers were the Hospital Universitari Dr. Josep Trueta-IAS (Girona, Spain), the Hospital Universitari de Bellvitge (L’Hospitalet de Llobregat, Spain), and the Consorci Hospitalari de Vic (Vic, Spain). The study protocol (clinical investigation code: RAID-CRC 20202015) was approved by the Clinical Research Ethics Committee of the three participating centers. Written informed consent was obtained from all participants. From 189 recruited subjects, 17 had to be excluded because of poor sample condition. Finally, 172 samples from asymptomatic subjects were used in the proof-of-concept study.

**Table 1:** Characteristics of patients included in the proof-of-concept (RAID-CRC 20202015) and clinical validation (GG-RAIDCRC-1002) studies, classified according to their diagnosis.

	Characteristics	CRC	AA	NAA	NC
**Proof of concept**	**n (%)**	11 (6.3)	67 (39.0)	38 (22.1)	56 (32.6)
	**Age (mean, range)**	61 (50–69)	61 (50–69)	60 (50–69)	59 (49–69)
	**Sex, female (%)**	6 (54.5)	19 (28.3)	15 (39.5)	37 (66.1)
**Clinical validation**	**n (%)**	19 (5.8)	124 (37.9)	85 (26.0)	99 (30.3)
	**Age (mean, range)**	61 (54–69)	61 (50–73)	61 (50–70)	58 (49–69)
	**Sex, female (%)**	6 (31.6)	52 (41.9)	42 (49.4)	53 (53.5)

CRC, colorectal cancer; AA, advanced adenoma; NAA, nonadvanced adenoma; NC, normal colonoscopy.

All subjects underwent colonoscopy to determine their colorectal status. According to endoscopic examination and pathology results, diagnosis was classified into four groups: normal colonoscopy (colonoscopy with no findings or with sigma and/or rectum hyperplastic polyps <10 mm), nonadvanced adenomas (NAAs; tubular adenomas <10 mm with low-grade dysplasia), AAs (adenomas ≥10 mm or with villous component or high-grade dysplasia, and pTis adenocarcinoma), and invasive CRC. Serrated lesions were classified in the NAA (lesions <10 mm without dysplasia) or AA (lesions ≥10 mm or with dysplasia) groups. Advanced neoplasms were defined as AA or invasive CRC. Individuals were also asked to answer a questionnaire to record clinical and epidemiologic data.

This proof-of-concept allowed us to develop an algorithm that discriminates between healthy subjects and those with AN. It was validated on an independent cohort consisting of 359 consecutive FIT-positive participants in the national CRC screening program (Table 1). Clinical validation was designed as a cross-sectional, multicenter study; the protocol (clinical investigation code: GG-RAIDCRC-1002) was approved by the Clinical Research Ethics Committee of Hospital Universitari Dr. Josep Trueta-IAS and Hospital Universitari de Bellvitge. Written informed consent was obtained from all study participants. According to the endoscopic findings, subjects were classified into the four groups previously mentioned. From the 359 recruited subjects, 32 had to be excluded from the study for different reasons: the subject did not collect the stool sample, the sample was incorrectly conserved, there was no colonoscopic diagnosis, and the sample condition was poor. Finally, 327 samples were used for the clinical validation of *RAID-CRC Screen*.

### Fecal sample collection

Participants were asked to collect a stool sample from one bowel movement in a sterile container before colonoscopy and prior to bowel cleansing. Samples were immediately frozen after deposition. Then, subjects brought samples to the hospital, where they were kept frozen at -20°C for short-term storage and stored at -80°C upon arrival at the GoodGut S.L. facilities in Girona (Spain).

### DNA extraction from stool samples

Genomic DNA was extracted from frozen fecal samples after homogenization using the NucleoSpin® Soil Kit (Macherey-Nagel GMbH & Co., Duren, Germany). The instructions of the manufacturer were followed, and the DNA was finally eluted in a 100 μl final volume of SE Elution Buffer and stored at -20°C until use. The DNA concentration was determined with Qubit fluorometric quantification (Thermo Fisher Scientific, Massachusetts, USA).

### qPCR assay for CRC biomarkers

The specific bacterial sequences targeted were classified into five different groups according to their characteristics: Eubacteria (EUB) as the total bacterial load [22]; B10 (best BLAST match *F*. *prausnitzii*), B46 (best BLAST match *S*. *variabile*), B48 (best BLAST match *Ruminococcus*, *Roseburia*, *Coprococcus*), FPRA [23], and *Roseburia intestinalis* (RSBI) as butyrate-producing bacterial biomarkers; GMLL, PTST, and BCTF as opportunistic pathogens usually found in the oral cavity or in the bowel of mammals; *Bacteroides thetaiotaomicron* (BCTT) as a saccharolytic species; and *Escherichia coli* (ECO) as a proinflammatory species [24].

Quantification of CRC-specific biomarkers was performed by preparing specific reactions for each biomarker using SYBR Green Master Mix (Promega, Madison, USA) or Probe Master Mix (Promega, Madison, USA). The sequences of the forward and reverse primers and probes (when applied) are described in S1 Table. Each reaction consisted of 10 μl containing 1× GoTaq qPCR Master Mix, between 150 nM and 300 nM of each primer and/or probe, and up to 10 ng of genomic DNA template. The species-specific primers were purchased from Macrogen (Macrogen, Seoul, South Korea). All qPCR was run on an AriaMx Real-time PCR System (Agilent Technologies, Santa Clara, USA). Thermal profiles were different according to the biomarker analyzed (S2 Table). A melting curve step was added to the end of each qPCR to verify the presence of the expected amplicon size as well as to control primer dimer formation. Data were collected and analyzed with Aria Software version 1.5 (Agilent Technologies, Santa Clara, USA). All samples were amplified in duplicate and were considered valid when the difference between threshold cycles (C_t_) was less than 0.6. A no-template control reaction and a standard curve (sequential dilutions from 10^7^ to 10^3^ genomic units/μL) were included in each PCR run to check qPCR efficiency.

In terms of qualitative analysis, the absence of a biomarker was considered if the obtained C_t_ value was not within its dynamic range, which is the interval of relative abundance in which a given bacterial marker can exist.

### Statistical analysis

Data normality was assessed through the Kolmogorov-Smirnov test. The nonparametric Kruskal-Wallis test was used to test differences in variables with more than two categories. Pairwise comparisons of subcategories of these variables were analyzed using a Mann-Whitney test. All comparisons using bacterial markers were performed between the relative abundances, which were normalized by the dynamic range of each bacterial marker.

Receiver operating characteristic (ROC) curve analysis was applied to determine the usefulness of each biomarker to distinguish among different colorectal neoplasia statuses. The accuracy of discrimination of each bacterial marker was measured by the area under the ROC curve. Since *RAID-CRC Screen* is a qualitative approach, the performance of the designed algorithm was calculated using likelihood ratios.

In the proof-of-concept study, machine learning was used to determine which combination of bacterial markers was capable of distinguishing subjects with AN from those with normal colonoscopy or NAA. The specific methodology consisted of an initial training iteration with 70% of 100 random partitions of the dataset and further validation with 30% of the remaining predictive models generated using 4 different machine learning algorithms (neural network, logistic regression, gradient boosting tree, and random forest). *RAID-CRC Screen* was eventually designed using a combination of six of the bacterial markers.

The clinical validation was performed in 359 subjects. Sample size was calculated using the online platform GRANMO v7.12, estimating the population by proportions, with a 95% confidence interval, an accuracy of +/- 5%, estimating a population percentage that is expected to be approximately 30%, with 10% repositions foreseen.

The sensitivity, specificity, PPV, negative predictive value, and accuracy of the designed algorithm were calculated using Epidat 3.1 software (SERGAS, Xunta de Galicia, Spain).

All remaining statistical analyses were performed using the SPSS 23.0 statistical package (IBM, NYC, USA). Significance levels were established for p-values ≤ 0.05.

## Results

### Proof-of-concept study

#### Fecal bacterial markers in neoplasia progression

**Fig 1:**
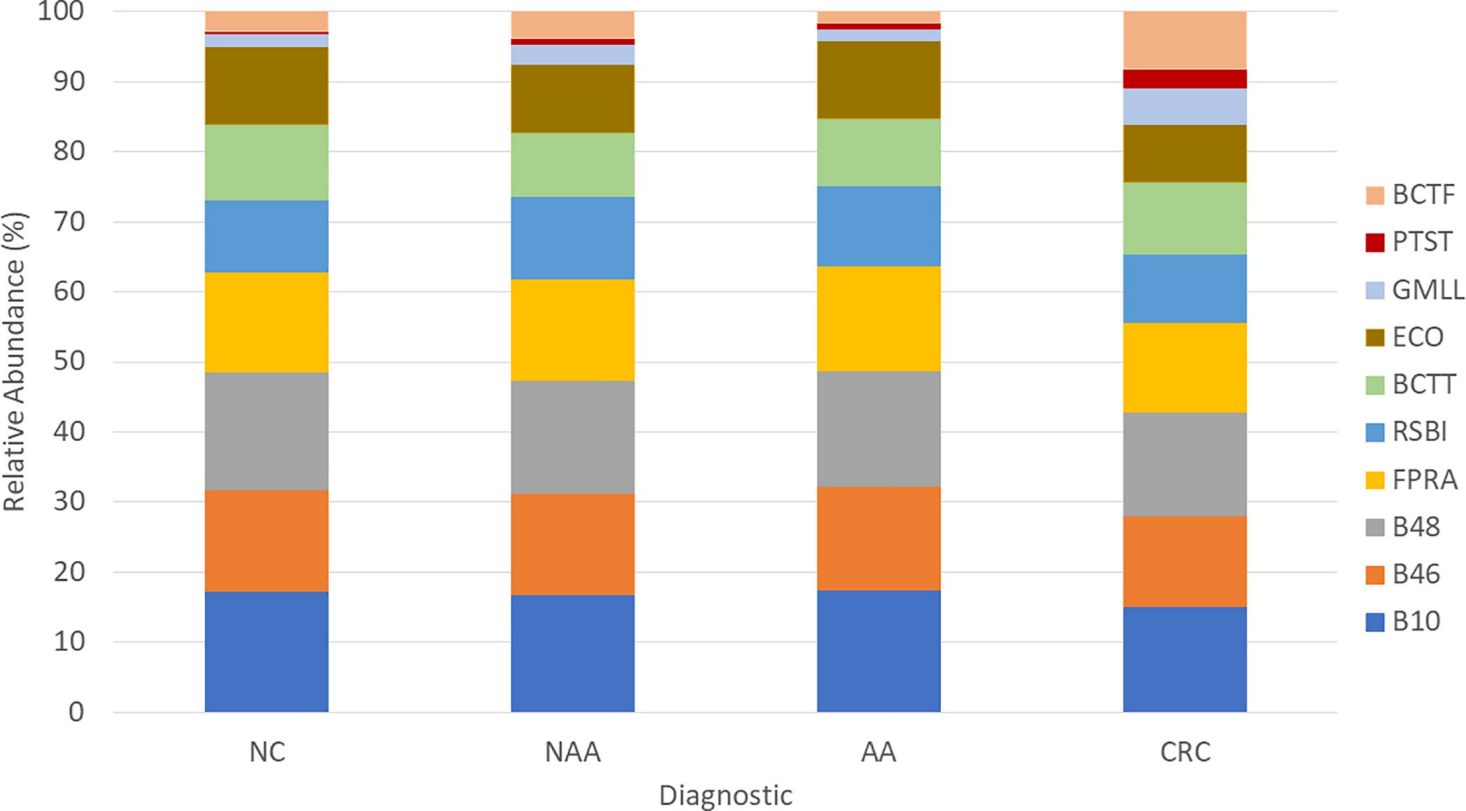
Relative abundance of the analyzed bacterial markers in subjects with normal colonoscopy (NC), nonadvanced adenoma (NAA), advanced adenoma (AA), and colorectal cancer (CRC). *F*. *prausnitzii* (FPRA), *G*. *morbillorum* (GMLL), *P*. *stomatis* (PTST), *B*. *fragilis* (BCTF), *B*. *thetaiotaomicron* (BCTT), *R*. *intestinalis* (RSBI) and *E*. *coli* (ECO)).

In the proof-of-concept study, stool samples were used to evaluate the relative abundance of each bacterial marker according to each diagnosis (Fig 1). Regardless of the colonoscopy result, the most prevalent bacterial species were butyrate producers (B10, B46, B48, FPRA, and RSBI), with relative abundance values of 16.7%, 14.2%, 16.1%, 14.1%, and 10.8%, respectively. Compared to healthy individuals, subjects with either NAA or AA showed a higher absolute abundance of B10, B46 and FPRA (p = 0.018, p = 0.004, and p = 0.023, respectively). PTST and BCTF absolute abundance was significantly higher in the CRC population than in individuals with normal colonoscopy (p = 0.002 and p = 0.017, respectively). Although there were no significant differences, a tendency could be observed in GMLL, as it showed a higher absolute abundance in CRC patients (p = 0.073); similarly, PTST and RSBI absolute abundances were higher in the presence of either NAA or AA (p = 0.056 and 0.060, respectively). Regarding ECO, no significant differences were observed among the studied groups.

On the one hand, higher absolute abundances of opportunistic pathogens (GMLL, PTST, and BCTF) were observed in CRC patients with respect to healthy subjects (p = 0.073, p = 0.002, and p = 0.017, respectively). In terms of prevalence, these bacterial markers were found more often in patients with CRC (GMLL, 75.0%; PTST, 50.0%; and BCTF, 66.6%) than in healthy subjects (GMLL, 40.8%; PTST, 11.1%; and BCTF, 31.3%). On the other hand, when subjects with normal colonoscopy were compared to patients with AN, three butyrate-producing bacteria (B10, B46, and FPRA) showed significant differences in their absolute abundance (p = 0.035, p = 0.030, and p = 0.042, respectively).

#### *RAID-CRC Screen* algorithm development

The development of the *RAID-CRC Screen* algorithm was aimed at reducing false-positive results for AN among FIT-positive subjects while maintaining 100% sensitivity for CRC. FPRA, B46, and B10 were the only bacterial markers that showed significant differences between subjects with normal colonoscopy or NAA and those with AN within the FIT-positive population; however, they did not provide the desired sensitivity values. An algorithm based on the combination of FPRA and B46 with three other bacterial species (B48, GMLL, and BCTF) and the total bacterial load exhibited a sensitivity of 94.8%, a specificity of 26.1%, a PPV of 50.0%, and a negative predictive value of 86.0% for AN (Table 2). More specifically, *RAID-CRC Screen* consists of 5 bacterial ratios: FPRA/EUB, B46/EUB, B48/EUB, GMLL/EUB, and BCTF/EUB applied to the FIT-positive population. While FIT 20 μg/g led to 94 false-positive results for AN detection, *RAID-CRC Screen* reduced this value to 62, implying a reduction of 34% in the false-positive rate. Notably, developers focused on sensitivity for AN, which reached 94.8%.

**Table 2:** Diagnostic performance of *RAID-CRC Screen* in the proof-of-concept study (n = 172).

Most advanced finding	Groups used for calculating specificity	Sensitivity (%)	Specificity (%)	PPV (%)	NPV (%)
Colorectal cancer (n = 11)	NAA+NC	100.0	28.0	13.0	100.0
Advanced adenoma (n = 67)	NAA+NC	94.0	26.0	49.0	88.0
Advanced neoplasia (n = 78)	NAA+NC	94.8	26.1	50.0	86.0

NC, normal colonoscopy; NAA, nonadvanced adenoma; PPV, positive predictive value; NPV, negative predictive value.

### Clinical validation study

When *RAID-CRC Screen* was scaled up to the 327 FIT-positive individuals of the validation cohort, sensitivities of 94.7%, 82.3%, and 83.9% were obtained for the detection of CRC, AA and AN, respectively (Table 3). Specificities were 16.3% among participants with NAA or normal colonoscopy and 18.2% among those with negative results on colonoscopy (Table 3). In this validation study, the algorithm detected 30 true negative subjects but generated 23 false negatives from the 327 FIT-positive individuals, 22 of them with AA and 1 with CRC. More importantly, while using FIT 20 μg/g, there were 184 false-positive results for AN, and *RAID-CRC Screen* reduced this figure to 154, which implies a reduction of 16.3% in the false-positive rate.

**Table 3:** Diagnostic performance of *RAID-CRC Screen* in the validation study (n = 327).

Most advanced finding	Groups used for calculating specificity	Sensitivity (%)	Specificity (%)	PPV (%)	NPV (%)
Colorectal cancer (n = 19)	NC+NAA	94.7	16.3	10.5	96.8
	NC	94.7	18.2	18.2	94.7
Advanced adenoma (n = 124)	NC+NAA	82.3	16.3	49.8	57.7
	NC	82.3	18.2	55.7	45.0
Advanced neoplasia (n = 143)	NC+NAA	83.9	16.3	43.8	56.6
	NC	83.9	18.2	59.7	43.9

NC, normal colonoscopy; NAA, nonadvanced adenoma; PPV, positive predictive value; NPV, negative predictive value.

## Discussion

Early detection of CRC is crucial for reducing its incidence and mortality. The European Union recommends population-based, organized screening for CRC using evidence-based methods with quality assurance of the entire screening process [25]. The best CRC screening strategy has not yet been defined, but the most widely used strategy in Western countries is FIT, which shows 79.0% sensitivity and 99.0% negative predictive value for CRC detection [12]. The most common limitation of FIT-based CRC screening programs is false-positive results [26], as they lead to patient concerns, additional costs [27], and adverse events associated with unnecessary colonoscopies. In this study, we have defined and clinically validated a fecal bacterial signature that complements FIT by reducing its associated false-positive rate among FIT-positive participants.

**Fig 2:**
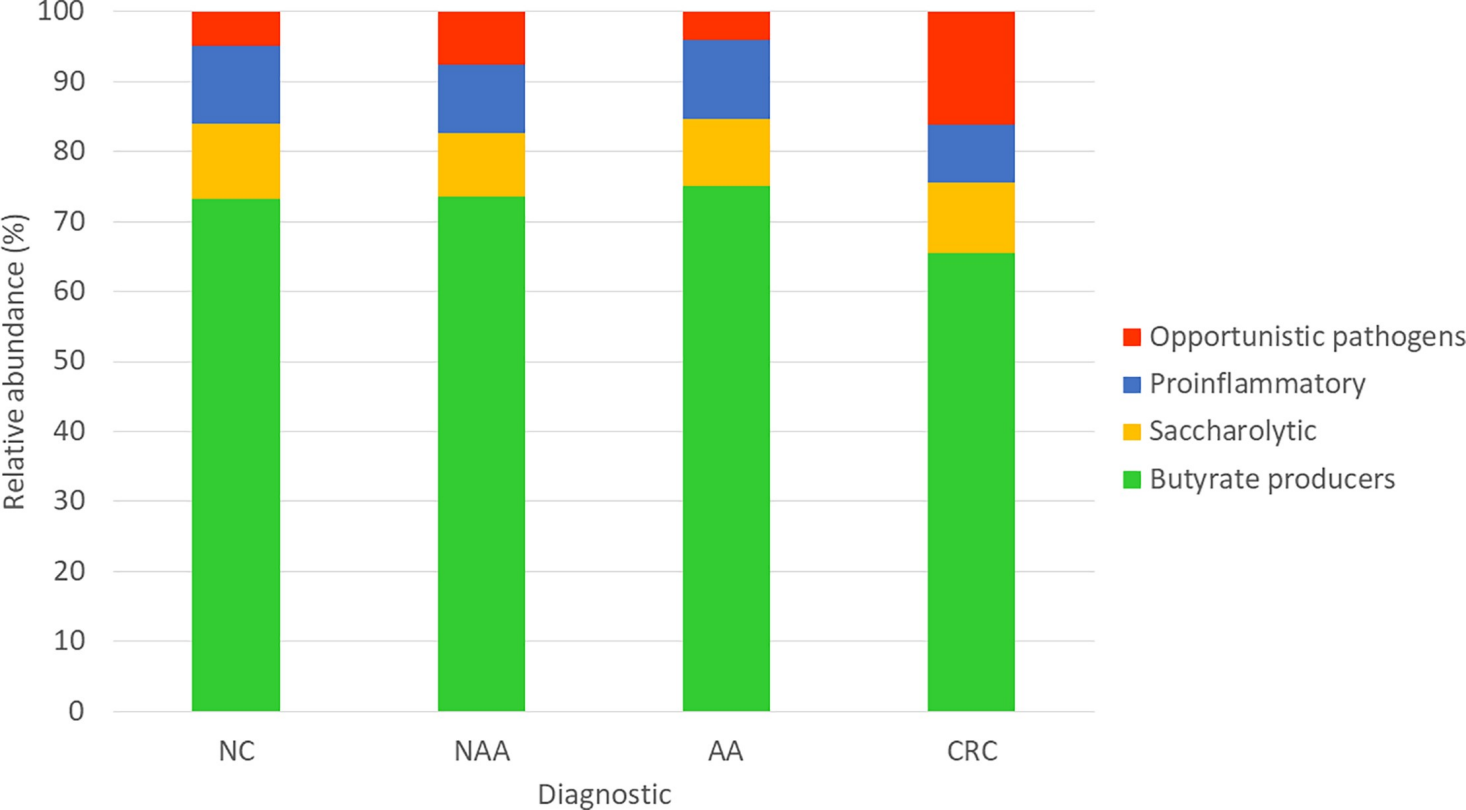
Relative abundance of the analyzed bacterial markers in subjects with normal colonoscopy (NC), nonadvanced adenoma (NAA), advanced adenoma (AA), and colorectal cancer (CRC). Butyrate-producing species (B10, B46, B48, FPRA, and RSBI); opportunistic pathogens (GMLL, PTST, BCTF); saccharolytic bacteria (BCTT); proinflammatory (ECO).

Different bacterial markers associated with CRC have been analyzed in a proof-of-concept study in subjects with colorectal neoplastic lesions at different stages: normal colonoscopy, NAA, AA and CRC. Our qPCR results indicate the existence of microbial dysbiosis in patients with CRC. The analyzed bacterial markers were classified according to their gut health-related phenotypes: butyrate-producing bacteria (B10, B46, B48, FPRA, RSBI), saccharolytic bacteria (BCTT), opportunistic pathogens (GMLL, PTST, BCTF), and proinflammatory bacteria (ECO) (Fig 2). The relative abundance of these microbiological groups differed significantly when healthy subjects were compared to CRC patients. Specifically, compared to subjects with normal colonoscopy, those with CRC exhibited a decreased abundance of butyrate-producing bacteria, which were replaced by pathogenic species. Unlike other studies, ECO did not display variations across different diagnostics [28]. Although BCTF has been considered a carcinogenesis driver, it was found in higher abundances solely in CRC [29,30].

The combination of these data with the FIT value was used to develop a screening method capable of discriminating between healthy subjects and patients with AN. The correct classification of subjects with CRC was prioritized; thus, a 100% sensitivity was sought. The algorithm was applied only to subjects with an FIT20 positive result (cut-off 20 μg hemoglobin/g feces) and was intended to maximize the reduction in FIT20 false-positive results while maintaining a high sensitivity for AN.

The defined microbiological signature combines five bacterial marker abundance ratios (FPRA/EUB, GMLL/EUB, B46/EUB, BCTF/EUB, B48/EUB). Our results showed that high FPRA/EUB, GMLL/EUB, and BCTF/EUB bacterial ratios correlated with the presence of AN. In contrast, low B46/EUB and B48/EUB bacterial ratios correlated with healthy individuals and AN, respectively. Notably, the use of the total bacterial load for data normalization is critical for controlling variables associated with qPCR, thus differentiating true biological changes from experimentally induced variation [31].

Clinical validation of *RAID-CRC Screen* confirmed high sensitivity (94.7%) and negative predictive values (96.8%) for CRC detection among FIT-positive individuals, respectively. Concerning the detection of AA, promising results were also obtained with a sensitivity of 82.3%. Altogether, *RAID-CRC Screen* reached a sensitivity and specificity of 83.9% and 18.2%, respectively, for the detection of AN. More importantly, the use of this new noninvasive tool achieved a reduction in unnecessary colonoscopies of up to 20% among subjects who tested positive for FIT. In addition, cancer screening programs seek methods with high negative predictive values since all affected individuals should be detected.

Although the two-step approach (FIT-positive result plus colonoscopy) is an efficient CRC screening strategy in an average-risk population [32,33], the use of bacterial signatures after testing positive in FIT may increase cost-effectiveness, as the number of unnecessary colonoscopies would be further reduced. Moreover, the use of fecal bacterial signatures might facilitate the implementation of CRC screening programs in resource-deprived regions, where colonoscopy availability is limited. The geographical origin of sample donors has been a matter of controversy that could impact the reproducibility of fecal bacterial signatures. This issue has been recently assessed by some research groups by performing meta-analyses with cohorts from different origins (USA, Germany, France, Italy, China, Canada, and Austria), and were able to identify reproducible microbiome markers in patients with CRC [19,34].

Since *RAID-CRC Screen* would be implemented in CRC screening organized programs, sample collection, conservation, and transport must be adapted to the existing circuits. Therefore, we have set up the detection of *RAID-CRC Screen* by qPCR from a unique sample taken with the FIT tube collector [20]. Thus, the availability of this screening test is linked to that of the general screening programs based on FIT. As for the cost, it will be slightly over the cost of FIT, considering further processing, but taking into account the reduction of colonoscopies it still will be more cost-effective than the current system.

Based on our results, we propose a three-step CRC screening strategy in which the bacterial signature (*RAID-CRC Screen*) is applied to FIT-positive individuals for a better selection of those who should undergo colonoscopy. This approach was associated with a significant reduction in false-positive FIT results among participants in a population-based, organized CRC screening program. Therefore, *RAID-CRC Screen* is being postulated as a new noninvasive tool for CRC screening, adding specificity and PPV to FIT while maintaining high sensitivity for AN.

## Supporting information

S1 Table Forward and reverse primers, and probe sequences used in this work. EUB, Eubacteria; GMLL, *G. morbillorum;* PTST, *P. stomatis;* BCTF, *B. fragilis;* BCTT, *B. thetaiotaomicron;* RSBI, *R. intestinalis;* FPRA, *F. prausnitzii;* ECO, *E. coli;* F, Forward primer; R, Reverse primer; PR, probe. FPRA probe was 5’-labelled with FAM (6-carboxyfluorescin) as the reporter dye and TAMRA was used as quencher dye at the 3’-end.
(RTF)

S2 Table qPCR conditions. EUB, Eubacteria; GMLL, *G. morbillorum;* PTST, *P. stomatis;* BCTF, *B. fragilis;* BCTT, *B. thetaiotaomicron;* RSBI, *R. intestinalis;* FPRA, *F. prausnitzii;* ECO, *E. coli;* NA, nonapplicable.
(DOCX)
