# Correction: New fecal bacterial signature for colorectal cancer screening reduces the fecal immunochemical test false-positive rate in a screening population

**DOI:** 10.1371/journal.pone.0334314

**Published:** 2025-10-10

**Authors:** Marta Malagón, Sara Ramió-Pujol, Marta Serrano, Joan Amoedo, Lia Oliver, Anna Bahí, Josep Oriol Miquel-Cusachs, Manel Ramirez, Xavier Queralt-Moles, Pau Gilabert, Joan Saló, Jordi Guardiola, Virginia Piñol, Mariona Serra-Pagès, Antoni Castells, Xavier Aldeguer, L. Jesús Garcia-Gil

There is an error in Table 2. The values in the Sensitivity (%), Specificity (%), PPV (%) and NPV (%) are incorrect. Please find the correct table below.

**Table 2:** Diagnostic performance of *RAID-CRC* Screen in the proof-of-concept study (n = 172).

Most advanced finding	Groups used for calculating specificity	Sensitivity (%)	Specificity (%)	PPV (%)	NPV (%)
Colorectal cancer (n = 11)	NAA + NC	100.0	34.0	15.1	100.0
Advanced adenoma (n = 67)	NAA + NC	95.5	34.0	50.8	91.4
Advanced neoplasia (n = 78)	NAA + NC	96.2	34.0	54.7	91.4
