# Supplementary material for: New fecal bacterial signature for colorectal cancer screening reduces the fecal immunochemical test false-positive rate in a screening population
Source: PLoS One. 2020 Dec 1;15(12):e0243158. doi: 10.1371/journal.pone.0243158 (PMC7707514; doi:10.1371/journal.pone.0243158)
Supplement: S1 Table — EUB, Eubacteria; GMLL, G. morbillorum; PTST, P. stomatis; BCTF, B. fragilis; BCTT, B. thetaiotaomicron; RSBI, R. intestinalis; FPRA, F. prausnitzii; ECO, E. coli; F, Forward primer; R, Reverse primer; PR, probe. FPRA probe was 5’-labelled with FAM (6-carboxyfluorescin) as the reporter dye and TAMRA was used as quencher dye at the 3’-end. (RTF) [file pone.0243158.s001.rtf]

S1 Table. Forward and reverse primers, and probe sequences used in this work. EUB, Eubacteria; GMLL, G. morbillorum; PTST, P. stomatis; BCTF, B. fragilis; BCTT, B. thetaiotaomicron; RSBI, R. intestinalis; FPRA, F. prausnitzii; ECO, E. coli; F, Forward primer; R, Reverse primer; PR, probe. FPRA probe was 5'-labelled with FAM (6-carboxyfluorescin) as the reporter dye and TAMRA was used as quencher dye at the 3'-end.
Target	Primers / Probe	Sequence 5' → 3'	Primer/Probe concentration	Ref	
EUB	EUB_F	ACT CCT ACG GGA GGC AGC AGT	200 nM	modified (22) 	
	EUB_R	GTA TTA CCG CGG CTG CTG GCA C			
B10	B10_F	CAA CAA GGT AAG TGA CGG C	300 nM	(21)	
	B10_R	CGC CTA CCT GTG CAC TAC TC			
B46	B46_F	TCC ACG TAA GTC ACA AGC G	300 nM	(21) 	
	B46_R	CGC CTA CCT GTG CAC TAC TC			
B48	B48_F	GTA CGG GGA GCA GCA GTG	300 nM	(21)	
	B48_R	GAC ACT CTA GAT GCA CAG TTT CC			
GMLL	GMLL_F	AAG AGT TCC AAG GCG TTC TC	150 nM	This study	
	GMLL_R	CCA TTT CAA GAT CCG CTT TCT ATT T			
PTST	PTST_F	AGG TTG ATG CTC TGA GTA GTA G	150 nM	This study	
	PTST_R	ATG AAT ACT AGC CTC TCC TCT TT			
BCTF	BCTF_F	TGA AAG CGT GCT CTT ACT ATT G	150 nM	This study	
	BCFT_R	TAT TGG CTG TTG TGC TTT GT			
BCTT	BCTT_F	AGT GAC CTG AAA GAA TCC TAA T	150 nM	This study	
	BCTT_R	GAC CGT CAA TAC CGA GAA AC			
RSBI	RSBI_F	GTG CCA GTA ACA GTC CAT ATT	150 nM	This study	
	RSBI_R	TAG CAA AGC AGA GTG GAA AG			
FPRA	FPRA_F
FPRA_R	TGT AAA CTC CTG TTG TTG AGG AAG ATA A
GCG CTC CCT TTA CAC CCA	300 nM
300 nM	(23)	
	FPRA_PR	6FAM-CAA GGA AGT GAC GGC TAA CTA CGT GCC AG-TAMRA	250 nM		
ECO	ECO_F	CAT GCC GCG TGT ATG AAG AA	300 nM	(24)	
	ECO_R	CGG GTA ACG TCA ATG AGC AAA	300 nM		
	ECO_PR	6FAM-TAT TAA CTT TTA CTC CCT TCC TCC CCG CTG AA-TAMRA	100 nM		
