# Supplementary material for: New fecal bacterial signature for colorectal cancer screening reduces the fecal immunochemical test false-positive rate in a screening population
Source: PLoS One. 2020 Dec 1;15(12):e0243158. doi: 10.1371/journal.pone.0243158 (PMC7707514; doi:10.1371/journal.pone.0243158)
Supplement: S2 Table — EUB, Eubacteria; GMLL, G. morbillorum; PTST, P. stomatis; BCTF, B. fragilis; BCTT, B. thetaiotaomicron; RSBI, R. intestinalis; FPRA, F. prausnitzii; ECO, E. coli; NA, nonapplicable. (DOCX) [file pone.0243158.s002.docx]

**S2 Table**. **qPCR conditions**. EUB, Eubacteria; GMLL, G. morbillorum; PTST, P. stomatis; BCTF, B. fragilis; BCTT, B. thetaiotaomicron; RSBI, R. intestinalis; FPRA, F. prausnitzii; ECO, E. coli; NA, nonapplicable.

| **Bacterial markers** | **Total cycles** | **Denaturing** | | **Annealing and Extension** | | **Melting curve** | |
| --- | --- | --- | --- | --- | --- | --- | --- |
|  |  | **Tª (ºC)** | **Time (min)** | **Tª (ºC)** | **Time (min)** | **Tª (ºC)** | **Time (min)** |
| EUB | 40 | 95 | 10:00 | 95  54 | 00:15  01:00 | 95  55  95 | 01:00  00:30  00:30 |
| B10, B46, B48 | 40 | 95 | 10:00 | 95  62 | 00:15  00:45 | 95  55  95 | 01:00  00:30  00:30 |
| GMLL, PTST, BCTT, RSBI | 40 | 95 | 10:00 | 95  60 | 00:15  01:00 | 95  55  95 | 01:00  00:30  00:30 |
| BCTF | 40 | 95 | 10:00 | 95  55  72 | 00:15  00:30  01:00 | 95  55  95 | 01:00  00:30  00:30 |
| FPRA, ECO | 40 | 50  95 | 02:00  10:00 | 95  60 | 00:15  01:00 | NA | NA |
